# The Effect of SGLT2 Inhibitor Dapagliflozin on Serum Levels of Apelin in T2DM Patients with Heart Failure

**DOI:** 10.3390/biomedicines10071751

**Published:** 2022-07-20

**Authors:** Alexander A. Berezin, Ivan M. Fushtey, Alexander E. Berezin

**Affiliations:** 1 Internal Medicine Department, Zaporozhye Medical Academy of Postgraduate Education, 69096 Zaporozhye, Ukraine; lunik.mender@gmail.com (A.A.B.); zmapo15@gmail.com (I.M.F.); 2 Internal Medicine Department, Zaporozhye State Medical University, 69035 Zaporozhye, Ukraine

**Keywords:** type 2 diabetes mellitus, heart failure, dapagliflozin, apelin, natriuretic peptides

## Abstract

Apelin is a multifunctional peptide that plays a pivotal role in cardiac remodeling and HF manifestation because of counteracting angiotensin-II. We hypothesized that positive influence of sodium-glucose co-transporter-2 (SGLT2) inhibitor on cardiac function in T2DM patients with HF might be mediated by apelin and that its levels seem to be a target of management. A total of 153 type 2 diabetes mellitus (T2DM) patients with II/III HF NYHA class and average left ventricular (LV) ejection fraction (EF) of 46% have been enrolled and treated with dapagliflosin. The serum levels of apelin and N-terminal brain natriuretic pro-peptide (NT-proBNP) were measured at baseline and over a 6-month period of dapagliflosin administration. We noticed that administration of dapagliflozin was associated with a significant increase in apelin levels of up to 18.3% and a decrease in NT-proBNP of up to 41.0%. Multivariate logistic regression showed that relative changes of LVEF, LA volume index, and early diastolic blood filling to longitudinal strain ratio were strongly associated with the levels of apelin, whereas NT-proBNP exhibited a borderline significance in this matter. In conclusion, dapagiflosin exerted a positive impact on echocardiographic parameters in close association with an increase in serum apelin levels, which could be a surrogate target for HF management.

## 1. Introduction

Type 2 diabetes mellitus (T2DM) is the leading metabolic factor of mortality risk from cardiovascular disease (CVD) including heart failure (HF) [1]. Although T2DM doubles a risk of de novo HF, it remains completely unclear what factors influence the disease manifestation [2]. Experimental and clinical data unveil numerous pathogenetic mechanisms, which contribute to cardiac dysfunction in T2DM patients including those that directly relate to CVD (accelerating atherosclerosis, coronary artery disease, myocardial infarction, atrial fibrillation), cardiovascular risk factors (dyslipidemia, obesity, chronic kidney disease, smoking) and more (lipid toxicity, insulin resistance, oxidative and mitochondrial stress, altered tissue reparation, endothelial dysfunction, microvascular inflammation, skeletal muscle and adipose tissue dysfunction) [3,4,5]. Amongst these mechanisms biomechanical stress and neurohumoral activation that are strongly associated with adverse cardiac remodeling play a pivotal role in HF manifestation [5,6].

Current clinical guidelines and statements for HF, regardless of its phenotypes, identify some circulating biomarkers of cardiac remodeling as secondary potential therapeutic targets in HF patients [7,8]. These biomarkers are considered to be natriuretic peptides (NPs), such as brain NP, N-terminal brain natriuretic pro-peptide and mid-reginal atrial natriuretic peptide, because their potencies have been accurately established in various large clinical trials and meta-analyses [9,10,11,12]. However, other targets of the metabolic-inflammatory circuit in T2DM patients at higher risk of HF are considered to be promising and are under serious scientific discussion, because a discriminative potency of NPs seems not to be optimal for T2DM patients [13,14]. A new four pillar-based strategy of HF management asks what underlying mechanisms allow one of its component-sodium-glucose co-transporter-2 (SGLT2) inhibitors to reduce mortality and hospitalization for HF independently from T2DM presence and whether old circulating biomarkers such as NPs can be used to predict HF in T2DM patients treated with SGLT2 inhibitors [15].

Apelin is the common name for a group of endogenous multifunctional peptides, which are ligands for angiotensin-like receptor 1 (APJ) and distinguished by their biological half-life. Under physiological conditions, apelin is widely expressed in numerous tissues and is responsible for cell migration and proliferation, tissue repair, immune reaction, oxidative stress, angiogenesis and vasodilation [16]. Apelin was found to be a key player in cardiac protection because of diminishing effects of angiotensin II [17]. Total apelin and its isoform called apelin-13 demonstrated positive inotropic and vasodilation effects in both normal and failing hearts in animals [18]. In addition, it decreased both systolic and diastolic blood pressures in hypertensive rats [19]. Moreover, apelin improved diastolic and systolic function associated with myocardial fibrosis and cardiac myocyte apoptosis in animals with HF [20]. Numerous animal and clinical studies suggest that reduced levels of circulating apelin could be risk factor for HF, while an expression of its receptor was found to have been decreased in hypertrophic myocardium as well as in non-ischemic and ischemic tissues of kidneys [21,22]. Overall, the levels of apelin may be a promising indicator of cardiac remodeling and biomarker of effective management of HF [23]. Moreover, T2DM apelin peptides attenuated insulin resistance, improved glucose tolerance and reduced circulating fasting glucose [24]. However, the impact of SGLT2 inhibitors on circulating levels of apelin remains uncertain. We hypothesized that positive influence of SGLT2 inhibitors on cardiac function in T2DM patients with HF might be mediated by apelin and that its levels seem to be a target of HF management. The purpose of the study was to investigate the effect of SGLT2 inhibitor dapagliflosin on the levels of apelin in patients with T2DM with different phenotypes of HF.

## 2. Materials and Methods

### 2.1. Materials

#### 2.1.1. Study Design and Patient Population

**Figure 1:**
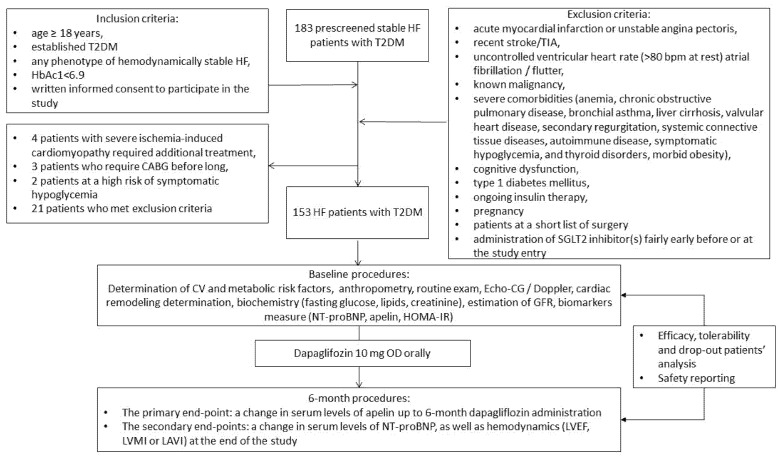
Flow chart of the study design. Abbreviations: T2DM, type 2 diabetes mellitus; CABG, coronary artery bypass grafting; TIA, transient ischemic attack; HF, heart failure; HOMA-IR, Homeostatic Assessment Model of Insulin Resistance.

The study is a multicenter open label non-randomized cohort investigation. The study design is shown in Figure 1. From an entire cohort of 183 T2DM patients we prospectively enrolled 153 patients with different phenotypes of HF aged 41 to 65 years who were recruited for the study from October 2020 to December 2021. The patients were treated in the following medical centers: the private hospital Vita-Centre (Zaporozhye, Ukraine), EliteMedService (Zaporozhye, Ukraine) and City Hospital #7 (Zaporozhye, Ukraine). The following inclusion criteria were used: age ≥ 18 years, established T2DM, hemodynamically stable HF (II-III NYNA functional classes), adequate control for hyperglycemia (HbAc1 < 6.9%) and written consent to participate in the study. Exclusion criteria were: acute myocardial infarction or unstable angina pectoris, recent stroke/transient ischemic attack (TIA), uncontrolled ventricular heart rate (>80 bpm at rest) due to atrial fibrillation/flutter, known malignancy, severe co-morbidities (anemia, chronic obstructive lung disease, bronchial asthma, liver cirrhosis, known valvular heart disease, symptomatic hypoglycemia, morbid obesity, congenital heart disease, systemic connective tissue diseases, autoimmune disease, cognitive dysfunction and thyroid disorders), type 1 diabetes mellitus, ongoing insulin therapy, pregnancy, patients on a short list for surgery and administration of SGLT2 inhibitor(s) fairly soon before or at the entry stage of the study.

#### 2.1.2. Treatment Identification

All patients were treated with SGLT2 inhibitor dapagliflozin (10 mg OD orally) that was added to the concomitant medication including metformin as a basic antidiabetic agent at beginning of the study. We used recommended HF therapy depending on the phenotypes of the conditions. Blood pressure lowering agents (ACE inhibitors/angiotensin-II receptor blockers (ARBs), calcium channel blockers, thiazide-like diuretics were used when needed to reach an optimal blood pressure control (office BP < 140/90 mmHg and/or average daily BP < 130/80 mm Hg). Beta-blocker in individually adjusted optimal daily dose along with mineralocorticoid receptor antagonist, ACE inhibitor or angiotensin receptor neprilysin inhibitor (ARNI) were administered to the patients with HF with reduced (HFrEF) and mildly reduced (HFmrEF) ejection fractions. Patients with HF with preserved ejection fraction (HFpEF) were not categorized in the specific treatment regime and took agents depending on their comorbidity status. Loop diuretics (furosemide, torasemide) were used when fluid retention was determined. Lipid-lowering medication (mainly rosuvastatin in average daily doses of 20–40 mg) were used in the majority of the patients without conventional contraindications. Antiplatelet drugs (acetylsalicylic acid 75 mg daily or clopidogrel 75 mg daily and oral anticoagulants (dabigatran 220–300 mg daily, rivaroxaban 10–20 mg daily) were used when needed to prevent atherothrombotic events and/or systemic thromboembolic complications. Because the clinical course of HF was stable, we did not change doses of other drugs during follow-up, although low-to-moderate loop diuretic could be modified temporary per request. The observation period was 6 months.

### 2.2. Methods

#### 2.2.1. Determination of CV Risk Factors and Co-Morbidities

Cardiovascular risk factors, such as hypertension, dyslipidemia, smoking habit, and T2DM, as well as stable coronary artery disease and chronic kidney disease have been evaluated in compliance with guidelines of the European Society of Cardiology (ESC) [24]. In order to determine HF during the study the guidelines that were valid at the time of enrollment were used [7,25].

#### 2.2.2. Anthropometric Measurements and Clinical Examinations

All patients enrolled in the study underwent general clinical and physical examination. We also measured office blood pressure (BP), heart rate, height, weight, waist circumference, hip-to-waist ratio (WHR) and body mass index (BMI).

#### 2.2.3. B-Mode Transthoracic and Doppler Examination

Echocardiography was performed with commercially available ultrasound systems comprising “GE Medical Systems” (GE, Freiburg, Germany), “Aplio 400” (Canon Medical Systems, Tochigi, Japan) and “Vivid E9” (GE-Vingmed, Horten, Norway). Standard echocardiographic measurements were obtained in accordance with the current guidelines of the American Society of Echocardiography/European Association of Cardiovascular Imaging [26]. Left ventricular (LV) ejection fraction (LVEF) was measured using Simpson method. Left atrial volume was directly measured and then left atrial volume index (LAVI) and E/e’ ratio were estimated. E/e’ ratio was estimated as a ratio between early mitral inflow velocity and mitral annular early diastolic velocity given as averaged septal and lateral e’. LV hypertrophy (LVH) was determined by conventional Echo criteria (LV mass/body surface area ≥125 g/m^2^ in male or ≥110 g/m^2^ in female) [26]. In addition, left ventricle myocardial mass index (LVMMI) was estimated according to the current recommendation [26].

#### 2.2.4. Estimating Glomerular Filtration Rate

Glomerular filtration rate (GFR) was calculated using the CKD-EPI formula [27].

#### 2.2.5. Insulin Resistance Determination

Insulin resistance was evaluated as Homeostatic Assessment Model of Insulin Resistance (HOMA-IR) using the conventional equation [28].

#### 2.2.6. Blood Sampling and Biomarker Measurement

Fasting blood samples were collected from an antecubital vein and placed in a tube. Within 30 min of blood collection, plasma was centrifuged for 15 min at 1600× *g* at 4 °C. Polled serum aliquots were then immediately stored in a refrigerator at ≤−70 °C until further analysis. We routinely used Roche P800 analyzer (Roche, Basel, Switzerland) to measure fasting levels of glycosylated hemoglobin (HbA1c), fasting glucose, insulin, total cholesterol (TC), low-density lipoprotein (LDL-C) cholesterol, high-density lipoprotein (HDL-C) cholesterol and triglycerides (TG). The serum levels of apelin and NT-proBNP were detected using commercially available ELISA kits (Elabscience, Houston, TX, USA) according to manufacturer’s instructions.

#### 2.2.7. Baselines and End-Point Measurements

The study measured the change in serum levels of apelin between baseline and 6 months after the beginning of dapagliflozin administration. The secondary measures comprised the change in brain natriuretic peptide (BNP), LVEF, LVMI or LAVI between baseline and 6 months after the dapagliflozin administration.

#### 2.2.8. Statistical Analysis

We used v.23 Statistical Packages for Social Sciences (SPSS; IBM, Armonk, NY, USA) software and v. 9 GraphPad Prism (GraphPad Software, San Diego, CA, USA) software for statistical analysis. The assumption of Gaussian distribution was tested using the Kolmogorov–Smirnov test. Continuous variables with normal distribution were characterized by mean ± standard deviation (SD), whereas continuous, non-normally distributed variables were specified by median (Me) and interquartile range (IQR). Categorical variables were expressed as frequencies and percentages. In order to compare groups between baseline and 6 months after the beginning of dapagliflozin administration we used paired *t* tests or Wilcoxon signed-rank test. Univariate logistic regressions were performed with the aim of elucidating possible associations of changes in apelin between baseline and 6 months after the start of dapagliflozin administration with clinical data (NYHA class), and relative changes of echocardiographic parameters (LVESV, LVEF, LAVI, and E/e’) and NT-proBNP. Then, all variables with *p* < 0.05 were transferred to and multivariate logistic regressions. For variables included in both regression models, we evaluated B coefficient, its standard deviation (SD), and *p* values. T-value and Variance Inflation Factors (VIF) were estimated for multiple logistic regression to examine multicollinearity. The intra-class correlation coefficient was used to determine both inter- and intra-observer reproducibility for apelin levels from 40 randomly selected patients using an identical cine-loop for each view. For all steps, *p* < 0.05 was considered statistically significant.

## 3. Results

### 3.1. Patients’ Characteristics

The baseline clinical and echocardiographic characteristics of T2DM patients with known HF are summarized in Table 1. The majority of the individuals were male (65.4%). Patients’ mean age was 52 years (41–64), body mass index (BMI) was 25.6 kg/m^2^, waist-to-hip ratio (WHR) was 0.85 units. The comorbidity signature comprised dyslipidemia (83%), arterial hypertension (86.3%), abdominal obesity (46.4%), smoking (41.2%) and left ventricular hypertrophy (80.3%). Stable coronary artery disease (CAD) and mild-to-moderate chronic kidney disease (22.9%) were detected in 32% and 22.9%, respectively. Microalbuminuria was found in 30.7% individuals. Average LV ejection fraction (LVEF) was 46% (39–54%), and 103 (67.3%)/50 (23.7%) patients had II/III HF NYHA class, respectively. Therefore, the patients were qualified as having HFpEF (31.4%), HFmrEF (32%) and HFrEF (36.6%).

### 3.2. Changes in Serum Levels of Apelin in Comparison with NT-proBNP during Dapagliflozin Administration

**Figure 2:**
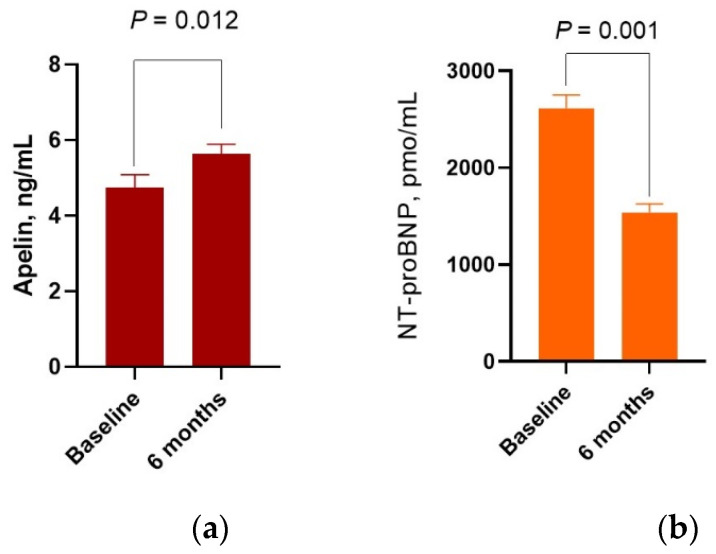
Bar graphs at baseline and 6 months after dapagliflozin administration, showing significant increase in serum levels of apelin (**a**) and decrease in NT-proBNP (**b**).

Over the 6-month period after initial prescription of SGLT2 inhibitor dapagliflozin the levels of apelin exhibited a significant growth up to 18.3% (from 4.75 [25–75% IQR = 2.84–7.32] ng/mL to 5.62 ng/mL [25–75% IQR = 3.94–7.32], *p* = 0.012) (Figure 2a). The circulating levels of NT-proBNP were found to be significantly reduced from 2615 (25–75% IQR = 1380–3750) pmol/mL to 1542 (25–75% IQR = 970–2075) pmol/mL (Δ% = −41.0%, *p* = 0.001) between the baseline and 6th month after dapagliflozin administration (Figure 2b).

**Figure 3:**
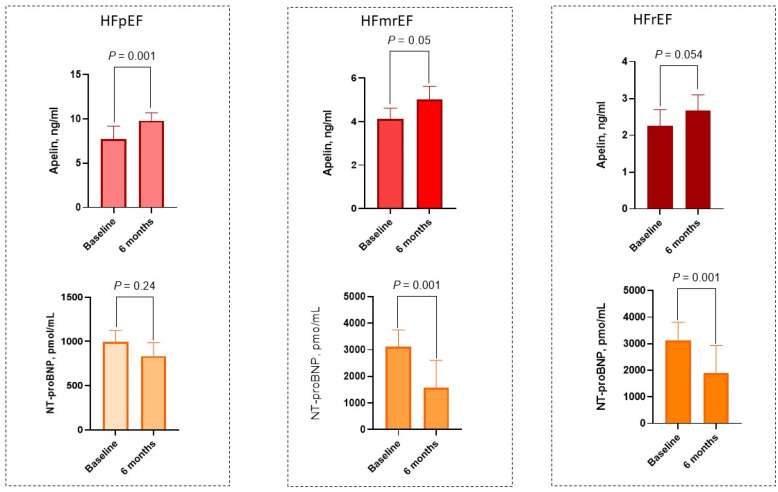
Bar graphs at baseline and 6 months after dapagliflozin administration, showing changes in serum levels of apelin NT-proBNP depending on the phenotypes of HF. Abbreviations: HFpEF, heart failure with preserved ejection fraction; HFmrEF, heart failure with mildly reduced ejection fraction; HFrEF, heart failure with reduced ejection fraction.

However, depending on the phenotypes of HF the levels of these biomarkers were differently changed over observation period (Figure 3). The levels of apelin demonstrated a significant increase in patients with HFpEF from 7.74 (25–75% IQR = 6.31–8.25) ng/mL to 9.80 (25–75% IQR = 7.90–10.52) ng/mL (Δ% = 21.0%, *p* = 0.001), whereas in individuals with HFmrEF and HFrEF a trend toward a statistically borderline increase in the concentrations of the peptide up to 17.9% (from 4.12 [25–75% IQR = 3.90–5.75] to 5.02 [25–75% IQR = 4.28–5.93] ng/mL; *p* = 0.050) and 15.7% (from 2.25 [25–75% IQR = 1.80–2.54] to 2.67 [25–75% IQR = 2.23–3.10] ng/mL *p* = 0.054), respectively, was noticed.

The levels of NT-proBNP exerted significant decrease in patients with HFrEF from 3125 (25–75% IQR = 2540–3810) pmol/mL to 1890 (25–75% IQR = 955–2930) pmol/mL (Δ% =−39.5%, *p* = 0.001) and HFmrEF from 3115 (25–75% IQR = 2380–3750) pmol/mL to 1580 (25–75% IQR = 870–2610) pmol/mL (Δ% = −49.3%, *p* = 0.001), but not for individuals with HFpEF. The concentrations of NT-proBNP showed a trend to decrease from 988 (25–75% IQR = 745–1126) pmol/mL to 843 (25–75% IQR = 697–985) pmol/mL (Δ% = −14.7%, *p* = 0.24).

### 3.3. Changes in Clinical Data and Hemodynamics Characteristics during Dapagliflozin Administration

All clinical and hemodynamics characteristics of the patients at baseline and over 6-month interval of dapagliflozin administration are given in Table 2. In fact, the proportion of HF with II and III NYHA classes changed significantly over the treatment. There was a significant increase in the number of HF patients with II HF NYHA class and decrease in those had III HF NYHA class. In addition, we noticed a significant reduction of left ventricular end-systolic volume (LVESV), left ventricular myocardial mass index (LVMMI), left atrial volume index (LAVI), early diastolic blood filling to longitudinal strain ratio (E/e‘) along with an increase in left ventricular ejection fraction (LVEF). Changes in biochemistry profile in T2DM patients with HF during observation period were not detected.

### 3.4. Association of the Changes in Apelin Levels with Hemodynamics Characteristics after Administration of Dapagliflozin

We performed univariate and multivariate logistic regressions analysis to detect associations of the dynamics of apelin levels with other variables after administration of dapagliflozin (Table 3). Univariate logistic regression indicated that relative changes (Δ) of LVEF, LAVI, LVMMI, E/e’ and NT-proBNP was significantly associated with the levels of apelin over the observation period. Multivariate logistic regression showed that among these echocardiographic parameters LVEF, LAVI, and E/e’ were strongly associated with the changes of the levels of apelin, whereas NT-proBNP exhibited a borderline significance in this matter. Thus, the changes in the apelin levels seem to be an indicator of favorable modification of echocardiographic parameters.

We further established that all predictors that had been identified in multiple logistic regression analysis were mildly correlated. We found none of the pairwise correlations among ΔLVEF, ΔLAVI, ΔLVMMI, ΔE/e’ and ΔNT-proBNP were particularly strong (r < 0.30 in each pairwise case). Estimated VIFs seem to show that the strength of the relationships between pertinent independent variables and any others was non-significant.

We then performed multivariate logistic regression analyses in HFpEF, HFmrEF, and HFrEF patients separately in which we included the variables that had significantly predicted changes of apelin levels (Table 4).

We noticed that ΔLVEF was an independent factor for the apelin levels in patients with HFrEF/HFmrEF, but not those with HFpEF (Table 4). On the contrary, ΔLAVI and ΔLVMMI exhibited their predictive values for apelin mainly in HFpEF. Furthermore, ΔE/e’ predicted a change of apelin levels regardless of HF phenotype.

### 3.5. Reproducibility of Apelin

The evaluation of the reproducibility of apelin was performed in comparison with NT-proBNP. The intra-class correlation coefficient for inter-observer reproducibility of NT-proBNP was 0.88 (95% confidence interval [CI] = 0.83–0.92), whereas the intra-class correlation coefficient for intra-observer reproducibility of apelin was 0.93 (95% CI = 0.91–0.96).

## 4. Discussion

The results of the study showed that SGLT2 inhibitor dapagliflozyn modified the levels of apelin depending on the phenotype of HF, exerting most meaningful effect in patients with HFpEF rather than HFmrEF or HFrEF. In contrast, dapagliflozin significantly reduced circulating levels of NT-proBNP in HFrEF and HFmrEF, but not in HFpEF. In addition, we established that relative changes of several echocardiographic parameters that reflected systolic (LVEF) and diastolic (LAVI and E/e’) function were associated with the dynamics of apelin levels. Therefore, the reproducibility of apelin was higher when compared with NT-proBNP. All these findings indicate that the levels of apelin may be regarded as a surrogate target during the management of HF in T2DM patients.

Several recent large clinical trials on different phenotypes of HF and exclusively showed a favorable effect of SGLT2 inhibitors on cardiac function. A possible explanation might be dynamic changes of counter regulation between tissue and circulating renin-angiotensin-aldosterone system (RAAS) [29]. Indeed, apelin acts a physiological antagonist of angiotensin-II, which is the main effector of RAAS [30]. Previous clinical studies among HFrEF patients have revealed that different components of pharmacological management of HF, such as angiotensin receptor-neprilysin inhibitor (ARNI), angiotensin-II receptor blockers, mineralocorticoid receptor antagonists, induced multidirectional changes in the level of apelin and NPs [31,32,33]. HFrEF patients with II/III NYHA classes, but not IV NYHA class individuals demonstrated an increased levels of apelin over the treatment period [33]. It has been noticed that there was no significant association between baseline apelin level (mainly apelin-12) and clinical parameters in HFrEF patients, whereas the dynamic changes of apelin over time were found to be significant predictors of the clinical course of the condition [31]. Moreover, acute administration of apelin in HF rapidly increased coronary blood flow, cardiac index, the maximum rate of rise in LV pressure and reduced peak and end-diastolic LV pressures, peripheral artery resistance, and mean arterial pressure [34]. At the same time, chronic administration of novel apelin receptor agonist AMG-986 did not demonstrate clinically meaningful pharmacodynamics effects in HFrEF [35]. However, there is a large volume of evidence that low levels of apelin may be a biomarker of adverse cardiac remodeling and untoward clinical course of HFrEF. Little is known about the clinical and predictive significance of apelin in HFpEF.

Overall, we hypothesized that dynamic changes of apelin levels controversially relate to cardiac hemodynamic performances in HFpEF and HFrEF and this interplay might be an attribute of altered metabolic homeostasis. The results of our study can be concisely explained if there is a protective effect of apelin in T2DM patients with known HF on cardiac structure and function is a result of uncoupling between the concentration of apelin and expression of its corresponding receptor (G protein-coupled receptor, APJ) on the surfaces of target cells including cardiac myocytes [35]. Indeed, apelin/APJ system alleviated mitochondrial dysfunction, which is a common phenomenon in T2DM-related and ischemia/hypoxia-induced cardiac myocyte injury [36]. In fact, interplay of apelin with APJ mediates a large number of molecular signaling pathways engaged in metabolic and functional recovery. The results of this cooperation are considered to include a significant increase myocardial capillary density and neoangigenesis, suppression of endoplasmic reticulum stress-induced cell apoptosis, and improvement of integrity of endothelium and cell membranes [36,37]. There has been debate whether sirtuin-depending activity of SGLT2 inhibitors might be an effective trigger for coupling of apelin/APJ system, thereby maintaining homeostasis [38]. Recent meta-analysis of 13 randomized clinical studies showed that revised adverse cardiac remodeling in HF patients may be a promising candidate explanation for the favorable clinical effects of SGLT2 inhibitors [39]. It is important to notice that the authors found a positive changes in global echocardiographic parameters, such as LVEDV, LVESV, LVEF, LAVI, and E/e’, in patients treated with SGLT2 inhibitors over the observation period, but not always corresponding to circulating biomarkers including NPs [39].

The results of our study seem to confirm the initial hypothesis that dapagliflosin, having a positive hemodynamic response in T2DM patients with HF, needs a surrogate biomarker with much better reproducibility than NT-proBNP. Moreover, NT-proBNP is considered not to be an optimal surrogate target for the therapy in T2DM with obesity, chronic kidney disease, and HFpEF due to its high serum variability, low diabetes risk and uncertainty in connection with echocardiographic parameters [40]. Moreover, in the general population the levels of NPs were inversely associated with incident T2DM and its progression, but their concentrations remained high in prognostic accuracy for all-causes death and hospitalization due to HF [40,41]. However, SGLT2 inhibitors reduced a number of serious HF events and improved clinical status in HF patients regardless of volume overload and consequently independently from the baseline level [42,43,44], pool of electrolytes and metabolic profile [45,46]. Overall, the cardiac protective effects of SGLT-2 inhibitors in patients with T2DM and HF are most likely attributable to multiple mechanisms, including peroxisome proliferator-activated receptor gamma- and perilipin-related metabolic effects, sirtuin-dependent anti-inflammatory and tissue protective effects, MAPK kinase/ERK dependent inhibition on adipogenesis and lipolysis [47,48].

Taken together, these findings do not support an idea about a dominant role of stimulating diuresis in reaching clinical benefits of SGLT2 inhibitors on the course of HF. It has been postulated that anti-inflammatory properties of SGLT2 inhibitors with cardiometabolic risk factors including T2DM can play a crucial role in improving survival and quality of life along with reducing the number of HF-related outcomes and hospital admissions [49]. If this proof of concept is correct, we need a new surrogate biomarker such as apelin to be added to NPs to accurately identify the earliest response of SGLT2 inhibition and predict effective management further. In this connection, the apelin-APJ system appears to be a useful surrogate target for management of HF in T2DM; knowledge of underlying molecular mechanisms and biological functions of apelin and its receptor APJ in the pathophysiology of both diseases may stimulate new approaches to management [50].

Although our study has several limitations, such as small sample size, open label design, enrollment of patients with stable HF with II/II NYHA class, a presence of full control for ventricular heart rate in atrial fibrillation, and good control for hyperglycemia, we believe that these findings can be useful to help discover a new algorithm to make the management of HF more effective and safe than conventionally used clinical-based strategy. We first report here that SGLT2 inhibitor dapagliflosin has a unique potential to modulate the levels of apelin in T2DM patients with HF and that this effect seems to be related to the phenotype of the condition. A large randomized clinical study in parallel groups of patients is needed to clearly elucidate the possible role of apelin levels as a surrogate target for HF management in T2DM patients.

## 5. Conclusions

In T2DM patients with different phenotypes of chronic HF, SGLT2 inhibition with dapagiflosin over a 6-month period of administration exerted a significant positive impact on echocardiographic parameters, such as LVEF, LAVI, and E/e’ along with a decrease in serum levels of NT-proBNP in close association with an increase in serum apelin levels. Future large clinical studies are needed to clearly elucidate whether the circulating levels of apelin can be a surrogate target of the HF management.

## Figures and Tables

**Table 1:** Baseline characteristics of T2DM patients (*n* = 153) included in the study.

Variables		Values
Demographics and anthropomorphic parameters		
	Age, year	52 (41–64)
	Male, *n* (%)	100 (65.4)
	BMI, kg/m^2^	25.6 ± 2.8
	Waist circumference, cm	85.1 ± 3.2
	WHR, units	0.85 ± 0.05
Comorbidities and CV risk factors		
	Dyslipidemia, *n* (%)	127 (83.0)
	Hypertension, *n* (%)	132 (86.3)
	Stable CAD, *n* (%)	49 (32.0)
	Smoking, *n* (%)	63 (41.2)
	Abdominal obesity,	71 (46.4)
	Microalbuminuria, *n* (%)	47 (30.7)
	LV hypertrophy, *n* (%)	123 (80.3)
	CKD 1–3 grades, *n* (%)	35 (22.9)
	Atrial fibrillation, *n* (%)	9 (5.90)
HF classification		
	HFpEF, *n* (%)	48 (31.4)
	HFmrEF, *n* (%)	49 (32.0)
	HFrEF, *n* (%)	56 (36.6)
	II/III HF NYHA class, *n* (%)	103 (67.3)/50 (32.7)
Hemodynamics		
	SBP, mm Hg	129 ± 6
	DBP, mm Hg	78 ± 5
	LVEDV, mL	161 (154–170)
	LVESV, mL	86 (80–93)
	LVEF, %	46 (39–54)
	LVMMI, g/m^2^	154 ± 5
	LAVI, mL/m^2^	39 (34–45)
	E/e’, unit	13.5 ± 0.3
Biomarkers		
	eGFR, mL/min/1.73 m^2^	75 ± 4.0
	HOMA-IR	7.95 ± 2.3
	Fasting glucose, mmol/L	5.62 ± 1.3
	HbA1c, %	6.59 ± 0.02
	Creatinine, mcmol/L	108.6 ± 8.5
	TC, mmol/L	6.43 ± 0.60
	HDL-C, mmol/L	0.97 ± 0.17
	LDL-C, mmol/L	4.38 ± 0.10
	TG, mmol/L	2.21 ± 0.17
	NT-proBNP, pmol/mL	2615 (1380–3750)
	Apelin, ng/mL	4.75 (2.84–7.32)
Concomitant medications		
ACEI, *n* (%)		72 (47.1)
	Ramipril 10 mg daily	10 (6.5)
	Ramipril 5–7.5 mg daily	6 (3.9)
	Ramipril 2.5 mg daily	3 (1.96)
	Perindopril 10 mg daily	41 (26.8)
	Perindopril 5 mg daily	12 (7.8)
ARB, *n* (%)		25 (16.3)
	Valsartan 320 mg daily	12 (7.8)
	Valsartan 160 mg daily	13 (8.5)
ARNI, *n* (%)		56 (36.6)
	Sacubitril/valsartan 97/103 mg OD	52 (34.0)
	Sacubitril/valsartan 97/103 mg twice per day	2 (1.30)
Beta-blocker, *n* (%)		136 (88.9)
	Bisoprolol 10 mg daily	33 (21.6)
	Bisoprolol 5–7.5 mg daily	31 (20.3)
	Bisoprolol 2.5 mg daily	4 (2.60)
	Nebivolol 10 mg daily	20 (13.1)
	Nebivolol 5–7.5 mg daily	12 (7.84)
	Carvedilol 50 mg daily	19 (12.4)
	Carvedilol 25–37.5 mg daily	17 (11.1)
I/f blocker, *n* (%)		21 (13.7)
	Ivabradin 10 mg daily	21 (13.7)
Calcium channel blocker, *n* (%)		27 (17.6)
	Amlodipine 10 mg daily	2 (1.3)
	Amlodipine 5 mg daily	25 (16.3)
MRA, *n* (%)		105 (68.6)
	Eplerenon 50 mg daily	56 (36.6)
	Eplerenon 25 mg daily	49 (32.0)
Loop diuretic, *n* (%)		132 (86.2)
	Furosemide > 160 mg weekly	50 (32.7)
	Furosemide < 160 mg weekly	42 (27.5)
	Torasemide 80–160 mg daily	12 (7.84)
	Torasemide < 80 mg daily	28 (18.3)
Antiplatelet, *n* (%)		135 (88.2)
	Acetylsalicylic acid 75 mg daily	86 (56.2)
	Clopidogrel 75 mg daily	49 (32.0)
Anticoagulant, *n* (%)		18 (11.8)
	Dabigatran 220–300 mg daily	9 (5.90)
	Rivaroxaban 10–20 mg daily	9 (5.90)
Anti-diabetics agents, *n* (%)		141 (92.2)
	Metformin 1000–3000 mg daily	94 (61.4)
	Metformin < 1000 mg daily	47 (30.7)
Statins, *n* (%)		151 (98.7)
	Rosuvastatin 40 mg daily	112 (73.2)
	Rosuvastatin 20–30 mg daily	39 (25.5)

Abbreviations: ACEI, angiotensin-converting enzyme inhibitor; angiotensin receptor neprilysin inhibitor (ARNI); CAD, coronary artery disease; CKD, chronic kidney disease; BMI, body mass index; DBP, diastolic blood pressure; E/e’, early diastolic blood filling to longitudinal strain ratio; GFR, glomerular filtration rate; HDL-C, high-density lipoprotein cholesterol; HFpEF, heart failure with preserved ejection fraction; HFmrEF, heart failure with mildly reduced ejection fraction; HFrEF, heart failure with reduced ejection fraction; LVEDV, left ventricular end-diastolic volume; LVESV, left ventricular end-systolic volume; LVEF, left ventricular ejection fraction; LVMMI, left ventricle myocardial mass index, left atrial volume index, LAVI; left atrial volume index; LDL-C, low-density lipoprotein cholesterol; MRA, mineralocorticoid receptor antagonist; OD, once per day; SBP, systolic blood pressure; TG, triglycerides; TC, total cholesterol; WHR, waist-to-hip ratio. Notes: data of variables are given as mean ± SD and median (25–75% interquartile range).

**Table 2:** Comparison of variables between baseline and 6 months after the administration of dapagliflozin.

Variables	Baseline	6 Month	Δ%	*p* Value
Clinical characteristics				
BMI, kg/m^2^	25.6 ± 2.8	24.1 ± 1.9	−4.30	0.11
II HF NYHA class, *n* (%)	103 (67.3)	122 (79.7)	+15.6	0.04
III HF NYHA class, *n* (%)	50 (32.7)	31 (20.3)	−24.8	0.04
Hemodynamics				
SBP, mm Hg	129 ± 6	127 ± 5	−1.60	0.21
DBP, mm Hg	78 ± 5	75 ± 6	−3.8	0.22
LVEDV, mL	161 (154–170)	158 (150–167)	−1.90	0.46
LVESV, mL	86 (80–93)	80 (76–85)	−7.00	0.04
LVEF, %	46 (39–54)	50 (44–57)	+8.70	0.05
LVMMI, g/m^2^	154 ± 5	141 ± 5	−8.40	0.02
LAVI, mL/m^2^	39 (34–45)	35 (31–39)	−10.3	0.04
E/e’, unit	13.5 ± 0.3	10.7 ± 0.5	−20.7	0.02
Biomarkers				
eGFR, mL/min/1.73 m^2^	75 ± 4.0	78 ± 3.0	+4.0	0.82
Fasting glucose, mmol/L	5.62 ± 1.3	4.90 ± 1.0	−12.8	0.24
HbA1c, %	6.59 ± 0.02	6.47 ± 0.03	−1.74	0.31
Creatinine, µmol/L	108.6 ± 8.5	112.5 ± 7.0	+3.50	0.28
TC, mmol/L	6.43 ± 0.60	6.31 ± 0.50	−1.90	0.42
HDL-C, mmol/L	0.97 ± 0.17	0.98 ± 0.15	+1.00	0.66
LDL-C, mmol/L	4.38 ± 0.10	4.34 ± 0.12	−5.20	0.43
TG, mmol/L	2.21 ± 0.17	2.15 ± 0.14	−2.71	0.56

Notes: data of variables are given mean ± SD and median (25–75% interquartile range) Abbreviations: DBP, diastolic blood pressure; E/e’, early diastolic blood filling to longitudinal strain ratio; GFR, glomerular filtration rate; HDL-C, high-density lipoprotein cholesterol; LVESV, left ventricular end-systolic volume; LVEF, left ventricular ejection fraction; LVMMI, left ventricle myocardial mass index, left atrial volume index, LAVI; left atrial volume index.

**Table 3:** Univariate and multivariate logistic regressions analysis for the association of apelin levels with NYHA class, and relative changes in hemodynamics and NT-proBNP.

Variables	Univariate Logistic Regression			Multivariate Logistic Regression				
	B Coefficient	SD	*p* Value	B Coefficient	SD	T Value	*p* Value	VIF
NYHA class	−0.89	0.22	0.42	-				
ΔLVESV	−2.01	0.76	0.05	−1.99	0.52	0.70	0.12	1.82
ΔLVEF	3.26	0.48	0.040	2.73	0.50	1.42	0.046	2.37
ΔLVMMI	−2.55	1.12	0.001	−2.10	1.08	−1.18	0.052	3.03
ΔLAVI	−6.13	1.57	0.001	−6.10	1.44	−2.44	0.001	2.94
ΔE/e’	−7.83	1.22	0.001	−7.83	1.22	−2.81	0.001	3.20
ΔNT-proBNP	−1.07	0.64	0.012	−0.88	0.63	0.56	0.050	3.95

Abbreviations: OR, odds ratio; CI, confidence interval; SD, standard deviation; Δ, a relative change in variable after 6-month administration of dapagliflozin; VIF, Variance Inflation Factor.

**Table 4:** Multiple regression analysis for the association of apelin levels after administration of dapagliflozin depending of phenotypes of HF.

Variables	B Coefficient	SD	T Value	*p* Value
HFpEF				
ΔLVEF	1.52	0.43	0.51	0.066
ΔLVMMI	−2.70	1.90	−2.33	0.040
ΔLAVI	−5.20	1.37	−1.26	0.024
ΔE/e’	−8.70	1.40	−3.60	0.001
HFmrEF				
ΔLVEF	3.55	0.74	3.90	0.042
ΔLVMMI	−2.36	0.81	−1.04	0.12
ΔLAVI	−4.90	0.64	−2.70	0.050
ΔE/e‘	−6.10	1.06	−2.78	0.044
HFrEF				
ΔLVEF	3.92	0.66	4.12	0.001
ΔLVMMI	−1.80	0.90	−1.18	0.054
ΔLAVI	−7.50	0.82	−3.55	0.001
ΔE/e‘	−7.20	1.15	−3.40	0.001

Abbreviations: SD, standard deviation; Δ, a relative change in variable after 6-month administration of dapagliflozin; E/e’, early diastolic blood filling to longitudinal strain ratio; HFpEF, heart failure with preserved ejection fraction; HFmrEF, heart failure with mildly reduced ejection fraction; HFrEF, heart failure with reduced ejection fraction; LVEF, left ventricular ejection fraction; LVMMI, left ventricle myocardial mass index, left atrial volume index, LAVI; left atrial volume index.

## Data Availability

Not applicable.
